# A mannitol/sorbitol receptor stimulates dietary intake in *Tribolium castaneum*

**DOI:** 10.1371/journal.pone.0186420

**Published:** 2017-10-12

**Authors:** Tomoyuki Takada, Ryoichi Sato, Shingo Kikuta

**Affiliations:** Graduate School of Bio-Applications and Systems Engineering, Tokyo University of Agriculture and Technology, Koganei, Tokyo, Japan; AgroParisTech, FRANCE

## Abstract

In insects, perception of chemical stimuli is involved in the acceptance or rejection of food. Gustatory receptors (Grs) that regulate external signals in chemosensory organs have been found in many insects. *Tribolium castaneum*, a major pest of stored products, possesses over 200 *Gr* genes. An expanded repertoire of *Gr* genes appears to be required for diet recognition in species that are generalist feeders; however, it remains unclear whether *T*. *castaneum* recognizes a suite of chemicals common to many products or whether its feeding is activated by specific chemicals, and whether its Grs are involved in feeding behavior. It is difficult to determine the food preferences of *T*. *castaneum* based on dietary intake due to a lack of appropriate methodology. This study established a novel dietary intake estimation method using gypsum, designated the TribUTE (*Tribolium* Urges To Eat) assay. For this assay, *T*. *castaneum* adults were fed a gypsum block without added organic compounds. Sweet preference was determined by adding sweeteners and measuring the amount of gypsum in the excreta. Mannitol was the strongest activator of *T*. *castaneum* dietary intake. In a *Xenopus* oocyte expression, TcGr20 was found to be responsible for mannitol and sorbitol responses, but not for responses to other tested non-volatile compounds. The EC_50_ values of TcGr20 for mannitol and sorbitol were 72.6 mM and 90.6 mM, respectively, suggesting that TcGr20 is a feasible receptor for the recognition of mannitol at lower concentrations. We used RNAi and the TribUTE assay to examine whether *TcGr20* expression was involved in mannitol recognition. The amounts of excreta in *TcGr20* dsRNA-injected adults decreased significantly, despite the presence of mannitol, compared to control adults. Taken together, our results indicate that *T*. *castaneum* adults recognized mannitol/sorbitol using the TcGr20 receptor, thereby facilitating the dietary intake of these compounds.

## Introduction

Feeding behavior in insects is comprised of several processes for recognizing chemical compounds, tasting, continuous feeding, and digestion [1]. Food-acceptance or food-rejection actions in insects are determined by non-volatile compounds such as carbohydrates and caffeine contained in host plants [2, 3]. Chemical compounds stimulate the gustatory receptors (Grs) located in external sensory organs. Stimulated Grs transmit electrochemical signals through sensory neurons to the sub-esophageal ganglion and brain, thereby regulating the feeding behaviors of insects [4, 5]. Feeding behaviors differ across diverse insect species [6]. For example, larvae of the silkworm *Bombyx mori* are specialist-feeders that depend solely on mulberry leaves for their growth [7]. The *B*. *mori* larvae perceive gustatory stimulants such as sucrose, inositol, morin, and β-sitosterol in the mulberry leaves using their maxillary palps [8]. *Myo*-inositol, an indispensable nutrient and a factor that prolongs feeding behavior in *B*. *mori* larvae, is recognized via BmGr10 expression in sensory organs [9]. Therefore, Grs in specialist feeders play a key role in detecting specific compounds. In contrast, the generalist feeder *Helicoverpa armigera* utilizes a wider range of host plants. Given that generalists feed on various plants and plant products, many types of non-volatile chemical compounds contained in foods may be sensed by various Grs-expressing sensory organs [10, 11].

Genome analysis of *Tribolium castaneum*, a major pest of grains, cereals, pasta, chocolate, and nuts [12, 13], indicates that it possesses 207 *Grs* genes [14]. Sixty-two *Gr* genes in the antennae and 69 *Gr* genes in mouthparts are highly expressed, and this species shows expression of many more types of *Grs* than other insect species [15]. This expanded Gr family likely plays a functional role for host selection. One pressing question is whether *T*. *castaneum* recognizes a suite of chemicals common to many products, or whether food selection is activated by specific chemicals. This question could be answered by investigating, dietary intake in *T*. *castaneum* exposed to an artificial diet composed of selected compounds. Dietary intake in *T*. *castaneum* has previously been examined using dried flour; however, this approach is limited for evaluating preferences as organic compounds in flour cannot be completely separated [16, 17]. Therefore, the establishment of a dietary intake assay based on an organic-free system would be advantageous. One commonly-used method involves the measurement of swallowed liquid food composed of sugar and water, such as in the CAFE (Capillary Feeder) assay [18]. However, this method is only applicable for sucking or licking insects. Because *T*. *castaneum* prefers dry products with a water content less than 12% [17, 19], the CAFE assay cannot be used for investigation of its dietary preferences. Thus, relatively little is known of the dietary intake of *T*. *castaneum* due to the lack of appropriate methods using dry compounds. In this study, a novel dietary intake estimation method using gypsum blocks without added organic compounds was developed. Since the gypsum eaten by *T*. *castaneum* adults is not digested and is eventually excreted as a waste product, the measurement of excreta enables the quantification of dietary intake.

Additionally, sweet preference can be determined by adding sweeteners to act as a stimulant for feeding behavior. Sweeteners can act as ligands to stimulate Grs; however, to date, only a few Grs have been identified as ligand-stimulated in the ectopic system of generalist feeders such as *T*. *castaneum*. For example, HarmGr4 of *H*. *armigera* responds to fructose in a *Xenopus* oocyte expression system [11]. Furthermore, members of the Gr43-like clade in the Gr family, which includes HarmGr4, have been found in various Diptera and Lepidoptera to act as receptors of fructose and *myo*-inositol [9, 20]. Since Gr43-like genes are also present in *T*. *castaneum*, these genes may be stimulated by any sugars/sugar alcohols in this species. Here, we postulated that sweeteners identified in a dietary intake assay might be potential ligands of Gr43-like genes in *T*. *castaneum*. This study demonstrates that ligands of TcGr20 belong to the Gr43-like gene family using an exogenous expression system with a two-voltage clamp assay. We also analyzed the role of these ligands *in vivo* using a combination of RNAi and a dietary intake assay.

## Materials and methods

### Insects

Red flour beetles (*Tribolium castaneum* Herbst.) were obtained from Sumica Technoservice Co. (Hyogo, Japan) and reared on whole-wheat flour (Pioneer-kikaku, Kanagawa, Japan) and yeast (Saf-instant^®^, Lesaffre, Marcq-en-Baroeul, France). They were maintained at 29 ± 1°C and 70% relative humidity under a 16 h light: 8 h dark cycle. Newly emerged pupae were collected and cultured to the adult stage. Adult beetles at 1 week after eclosion were used for dietary intake assays. To assess the effect of sexual differences in the dietary intake assay, we identified and separated male and female insects at the pupal stage; the dietary intake assay was again performed at 1 week after eclosion.

### Total RNA preparation and cDNA synthesis

Total RNA was isolated from the antennae and mouthparts of 20 adults using ISOGEN II (NIPPON GENE, Tokyo, Japan) following the manufacturer’s instructions. cDNA was synthesized from total RNA using ReverTra Ace^®^ (TOYOBO, Osaka, Japan) with Oligo-dT primers. The primer sequences are shown in S1 Table. The cDNA transcription reaction was performed at 42°C for 90 min to produce cDNA and 99°C for 5 min to denature the reverse transcriptase. Open reading frames (ORF) of *TcGr20*, *TcGr21*, *TcGr27*, *and TcGr28* were amplified from cDNA by PCR using a high-fidelity DNA polymerase, PrimeSTAR^®^ HS (TaKaRa Bio, Shiga, Japan), with specific primers containing restriction enzyme recognition sites at 5′ and 3′ ends of the ORFs (S1 Table). Kozak sequences were inserted before start codons to enhance translational efficiency. PCR amplification conditions were: 35 cycles of denaturation at 98°C for 30 s, annealing at 55°C for 15 s, and extension at 72°C for 70 s. *TcGr20*, *TcGr21*, *TcGr27*, and *TcGr28* were directly subcloned into the expression vector pT7XbG2 (DDBJ accession number, AB255037) and sequence analyses were performed by Eurofins Genomics (Tokyo, Japan) to confirm the correctness of the construct. Sequence data were analyzed using FinchTV sequence scanner software.

### Quantitative RT-PCR

Total RNA was isolated from male and female adults (n = 20) with ISOGEN II (NIPPON GENE) following the manufacturer’s instructions. Gene expression levels were examined in various tissues: antennae, heads (without antennae but including mouthparts), thorax, abdomen, and legs. First-strand cDNA was synthesized using a PrimeScript™ RT reagent Kit (TaKaRa Bio) following the manufacturer’s instructions. The quality and concentration of synthesized cDNAs were measured using a NanoPhotometer^®^ NP80 (Implen, München, Germany). Quantitative RT-PCR was performed using SYBR^®^ Premix *Ex Taq*™ II (Tli RNaseH Plus, TaKaRa Bio) with a StepOnePlus™ (Thermo Fisher Scientific, Carlsbad, CA) under the following conditions: a holding cycle at 95°C for 10 min, followed by 40 cycles at 95°C for 15 s 60°C for 1 min; a melting curve analysis was carried out at 95°C for 15 s and 60°C for 1 min to confirm the presence of nonspecific PCR reactions. Relative expression levels were calculated using the 2-*ΔΔ*Ct method. The *TcGr20* expression levels in tissues were normalized against the expression levels of *T*. *castaneum* ribosomal protein S3 (*RpS3*, NCBI accession: NM_001172392.1). *RpS3* was used as a housekeeping gene due to its expression stability in larval to adult stages [21, 22]. The primer sequences used for these analyses are shown in S3 Table.

### Capped RNA synthesis and two-electrode voltage clamp electrophysiology

The procedures for capped RNA (cRNA) synthesis followed those described in a previous study [9]. Briefly, cRNA was synthesized using an mMESSAGE mMACHINE^®^ T7 kit (Thermo Fisher Scientific) according to the manufacturer’s instructions and stored at ‒80°C until use. Oocytes were injected with cRNA and incubated in modified Barth's saline (MBS) buffer supplemented with 10 mg/mL penicillin and streptomycin for 3 days at 20°C [23]. Negative control oocytes were injected with water and therefore showed only endogenous receptor activities. Whole-cell current was recorded using a two-electrode voltage clamp in a perfusion system with Ringer’s solution [9]. The current was amplified with an OC-725C amplifier (Warner Instruments, Hamden, CT, USA) at a holding potential of ‒70 mV, low-pass filtered at 50 Hz, and digitized at 1 kHz. Data was acquired by software pCLAMP™ 10 (Molecular Devices, Sunnyvale, CA). Dose-dependent response curves were fitted to a Hill slope curve using Prism 6 software (GraphPad, San Diego, CA).

### Evaluation of dietary intake

We developed a novel feeding system for *T*. *castaneum* adults using gypsum that contained a near-zero amount of organic compounds instead of artificial diets and wheat, termed the “TribUTE (*Trib**olium* Urges To Eat)” assay. The artificial gypsum diet was composed of dry powder and water mixed in a ratio of 1.3:1 (w/w) and contained 200 mM sugars or sugar alcohol solutions. The gypsum mixture was allowed to solidify at 65°C for 48 h. *T*. *castaneum* adults were kept in cages at 25°C and starved for one week. A gypsum block (a cube with sides of approximately 5 mm) was provided to each adult beetle in a 24-well polystyrene microplate. The microplate was covered with aluminum foil to prevent the build-up of static electricity. The beetles were kept for 48 h at 25°C with the gypsum block. The gypsum consumed by the *T*. *castaneum* adults was eventually excreted without digestion as a waste product that could be measured. The excreta of beetles fed the artificial gypsum diet were collected in 200 μL microtubes using a thin silicon wire under a stereomicroscope. The excreta were then dissolved in 50 μL deionized water to remove sugars or sugar alcohols, and the gypsum precipitate was dried thoroughly at 65°C for 24 h. This gypsum precipitate was weighed using a microbalance (AT201, Mettler-Toledo, OH).

### RNA interference

To silence expression of the *TcGr20* gene in adults, we synthesized a double-stranded RNA (dsRNA) *in vitro* using a MEGAscript^®^ T7 RNAi kit (Thermo Fisher Scientific) according to the manufacturer’s instructions. The primers sequences are shown in S4 Table. The dsRNA (1 μg/μL) was kept at –80°C until use. A sample of dsRNA (150–200 nL) was injected between the internode of the head and thorax of adult beetles using a Nanoject II injection system (Drummond Scientific Company, Broomall, PA) on a cooling block at –10°C. Emerald luciferase (*Eluc*, TOYOBO) was used as a negative control. The NCBI *Tribolium* genome (ID:216) database was searched for similar sequences to *Eluc*. Since to *Eluc* and *TcGr20* of more than 22 bp could potentially function as false targets, these sequences were trimmed from the dsRNA regions. The dsRNA-injected adults were kept at 25°C. Adult beetles were used for the TribUTE assays and quantitative RT-PCR at 48 h after injections.

## Results

### Identification of feeding promoting stimulants in *T*. *castaneum*

**Fig 1:**
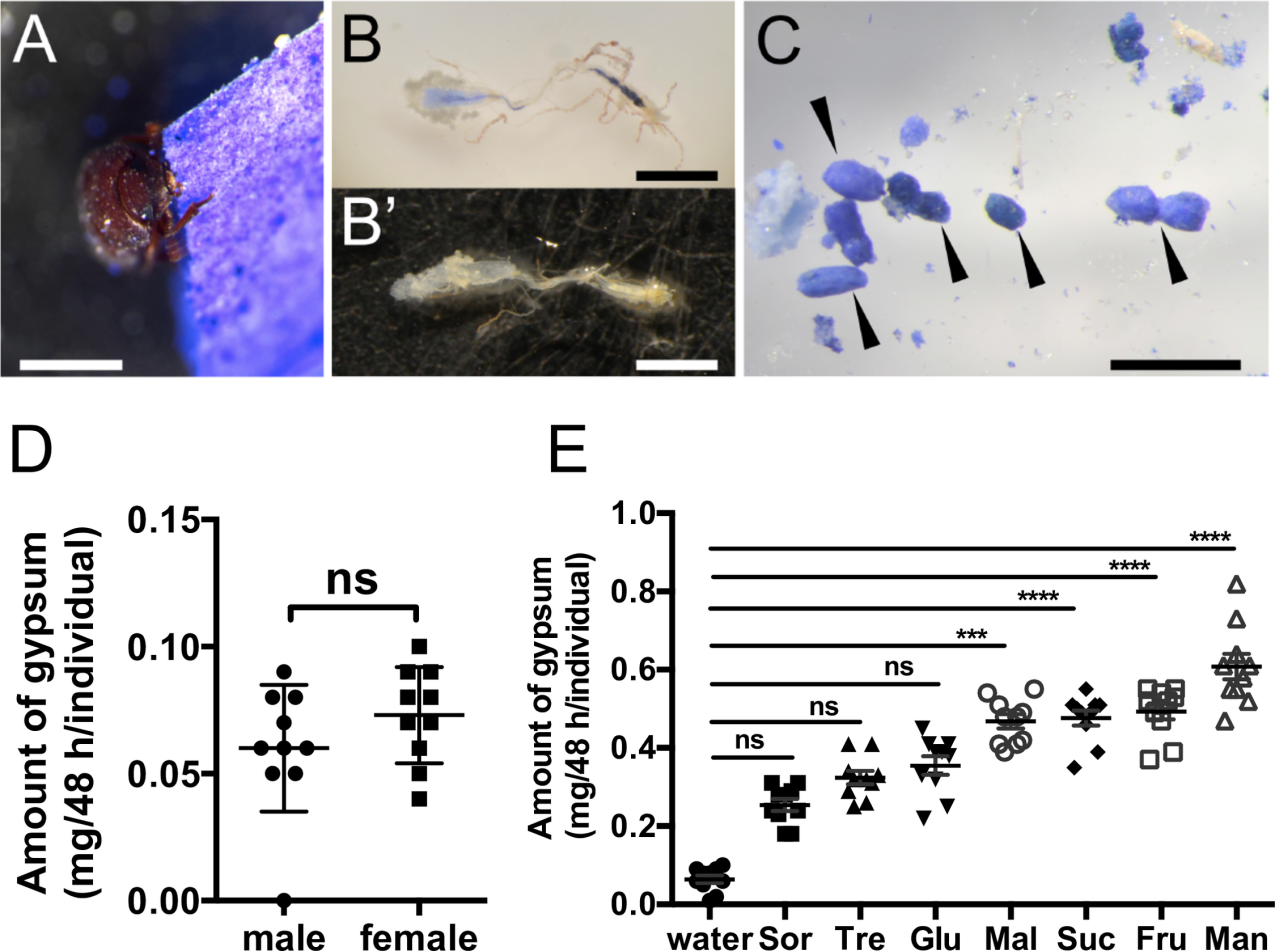
Establishment of feeding assay in *T*. *castaneum* adults using gypsum block. The starved *T*. *castaneum* adults show in a series of the behavior from recognition, eating to excretion the gypsum diet. Scale bars: 1 mm. A. The adult beetle bit gypsum. The gypsum was stained with Coomassie Brilliant Blue (CBB). B, B’. Digestive tract of adult beetle with the CBB staining gypsum (B) and without the CBB staining (B’). C. Excreta of the CBB staining gypsum by *T*. *castaneum* (arrowheads). D. The assessment of the effect of sexual differences in gypsum intake. *T*. *castaneum* adult beetles were individually fed on sweetener-free gypsum blocks for 48 h. The amount of excreta was measured using microbalance. Each plot represents the amount of excreta of adult beetles in individuals (n = 10). Standard error bars show S.E.M. Statistical analyses were performed Mann-Whitney U test (*P* = 0.26). “ns” not significant. Total numbers are 20. The results presented are representative of three separate experiments. E. The effect of gypsum intake with sugar or sugar alcohol mixture. Each sugar/sugar alcohol at 200 mM was contained in the gypsum block. *T*. *castaneum* adults fed on the artificial diet for 48 h. Each plot represents the amount of excreta of adult beetles in individuals (n = 10). Standard error bars show S.E.M. The results presented are representative of several separate experiments. Statistical analyses were performed Kruskal-Wallis test and the Dunn’s multiple comparison test (“***” *P* ≤ 0.001, “****” *P* ≤ 0.0001, “ns” not significant). Sorbitol, Sor; Trehalose. Tre; Glucose, Glu; Maltose, Mal; Sucrose, Suc; Fructose, Fru; Mannitol. Man. Total numbers are 80, and number of comparison family is 28.

We found that *T*. *castaneum* adults consumed gypsum block in the absence of organic compounds (Fig 1A). To discriminate gypsum excreta from conventional excreta derived from wheat flour, the gypsum blocks were stained with Coomassie Brilliant Blue (CBB) R-250 (Wako, Osaka, Japan) (Fig 1A). We examined the digestive tract of adults from the foregut to the anus under a microscope, and found that it showed partial staining with CBB (Fig 1B); no staining was present in the digestive tracts of beetles fed gypsum without CBB (Fig 1B’). The CBB staining of the blocks also resulted in the beetles producing excreta that could be identified (Fig 1C). Together, these findings showed *T*. *castaneum* adults fed on gypsum, and excreted it as a waste product, because they likely are recognizing the sugars added to the gypsum, and consequently they feed or not, it is uncertain whether insects recognize the gypsum itself or not. We found no evidence of sex-related differences in dietary intake of the gypsum (Fig 1D). We then examined the effects of feeding-promoting stimulants using gypsum blocks containing monosaccharides, disaccharides, or sugar alcohols. The amount of excreta produced by beetles fed gypsum with these additives was higher than that produced using sweetener-free gypsum (Fig 1E). Thus, *T*. *castaneum* recognized sugars and sugar alcohols as activators of food intake. We also examined the effects of adding 200 mM sorbitol, trehalose, or glucose to the gypsum and found that these compounds had a negligible and nonsignificant effect compared to gypsum without sweeteners (Fig 1E). Feeding the beetles with gypsum in the presence of 200 mM mannitol resulted in an increased level of excreta compared to the control, demonstrating that feeding behavior was stimulated by this sweetener. These findings indicate that mannitol prolonged feeding behavior in *T*. *castaneum*.

### Phylogenetic analysis of insect Grs

**Fig 2:**
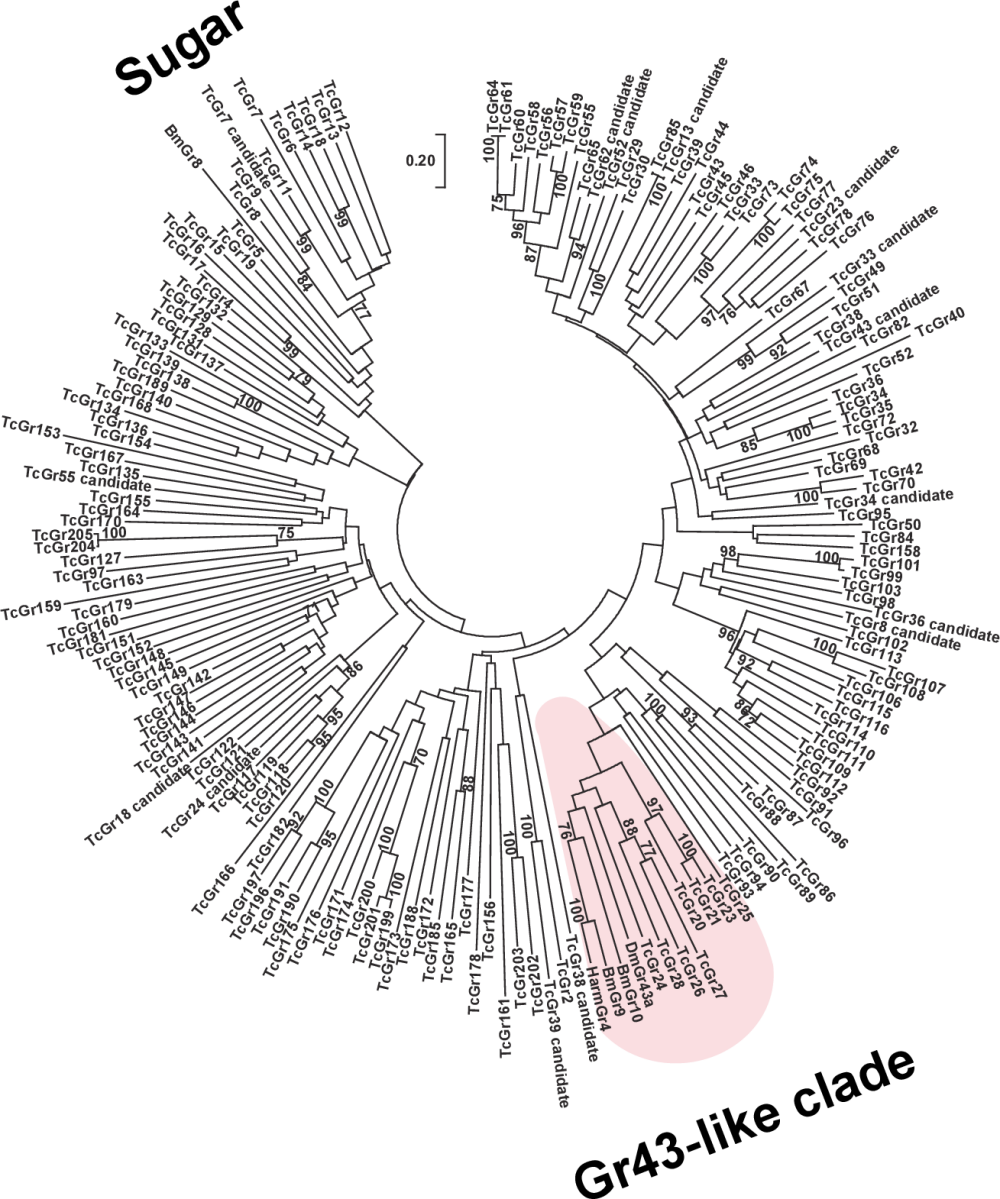
Phylogenetic analysis of deduced amino acid sequences of *T*. *castaneum* Gr genes. Amino acid sequence alignment was generated using ClustalW, and the unrooted tree of *T*. *castaneum* Grs was conducted by neighbor-joining method in MEGA ver. 7 [25]. The percentage of replicate trees are shown in the associated taxa clustered together in the bootstrap test of 500 replicates. The scale bar represents 0.2 substitutions per amino acid site. Amino acid sequences of *B*. *mori* and *T*. *castaneum* Grs were obtained from [9, 14], respectively. The amino acid sequences of fructose receptor, HarmGr4 from *Helicoverpa armigera*, DmGr43 derived from *Drosophila melanogaster*, were obtained from the NCBI public database.

Mannitol, a sugar alcohol, acts as a sweetener in vertebrate chemosensation [24], and other sugar alcohols are readily recognized by insect species. In *B*. *mori*, sugar alcohols such as *myo*/*epi*-inositol are recognized by the gustatory receptor BmGr10, which belongs to the Gr43-like clade [9]. We carried out a phylogenetic analysis based on amino acid sequences to identify homologous genes in *T*. *castaneum* belonging to the same clade as that encoding BmGr10. A phylogenetic tree of insect Grs was constructed using the neighbor-joining method (Fig 2). TcGr20–28 belonged to the clade that included the DmGr43a fructose receptor of *Drosophila melanogaster*, the BmGr9 fructose receptor and BmGr10 *myo*/*epi*-inositol receptors of *B*. *mori*, and the HarmGR4 fructose receptor of *H*. *armigera* [9, 11, 20]. On the basis of these results, we hypothesized that the mannitol receptors belonged to the TcGr20–28 groups.

### TcGr20 is a mannitol/sorbitol receptor

**Fig 3:**
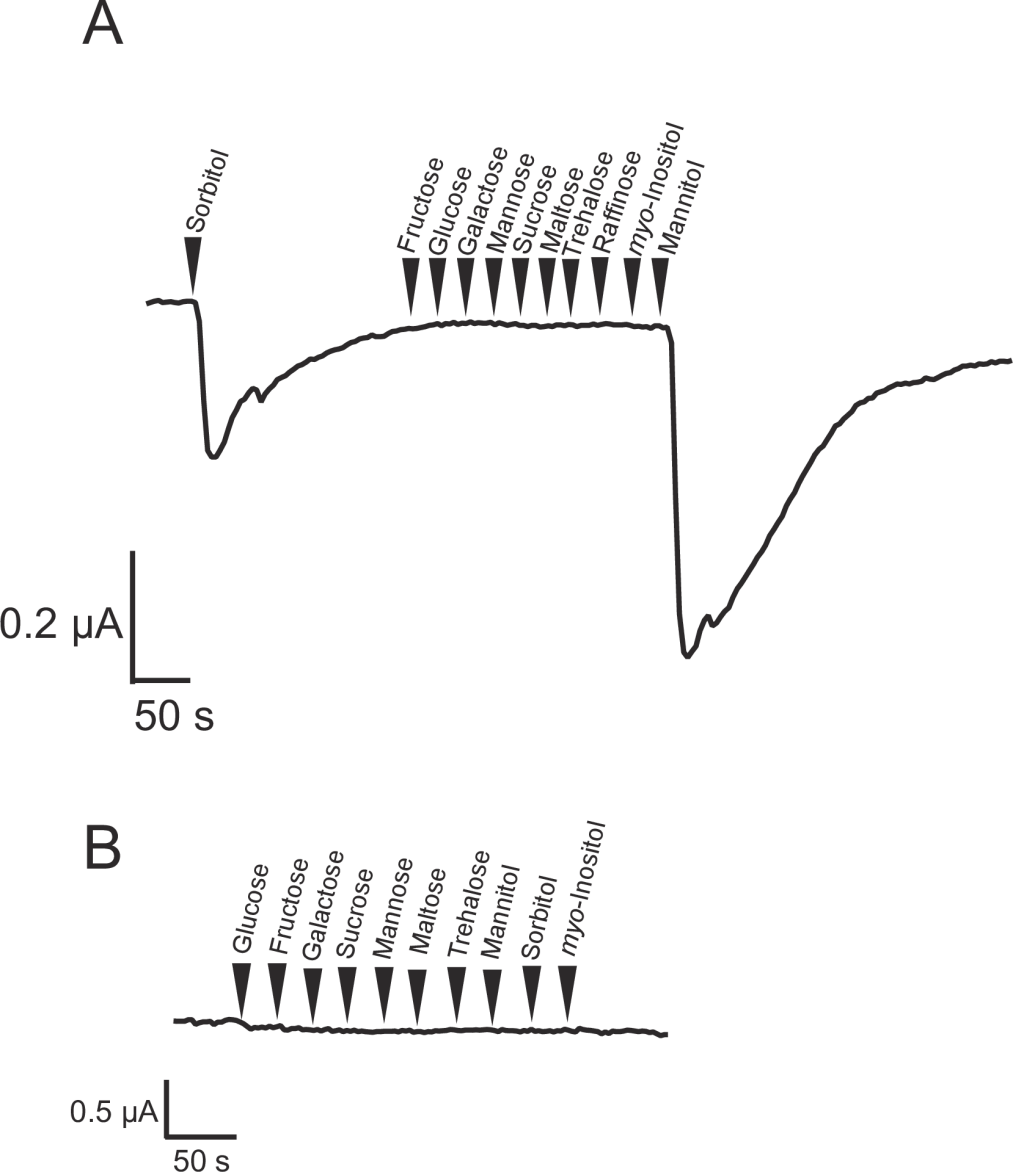
Current recording of *Xenopus* oocytes expressing TcGr20. A. Inward current response of *Xenopus* oocytes expressing TcGr20 to candidate tastants (arrowheads). Tastants were tested at 200 mM. B. The current of water-injected oocytes to same tastants were also recorded. The current data are representative of recordings independently performed in several times.

**Fig 4:**
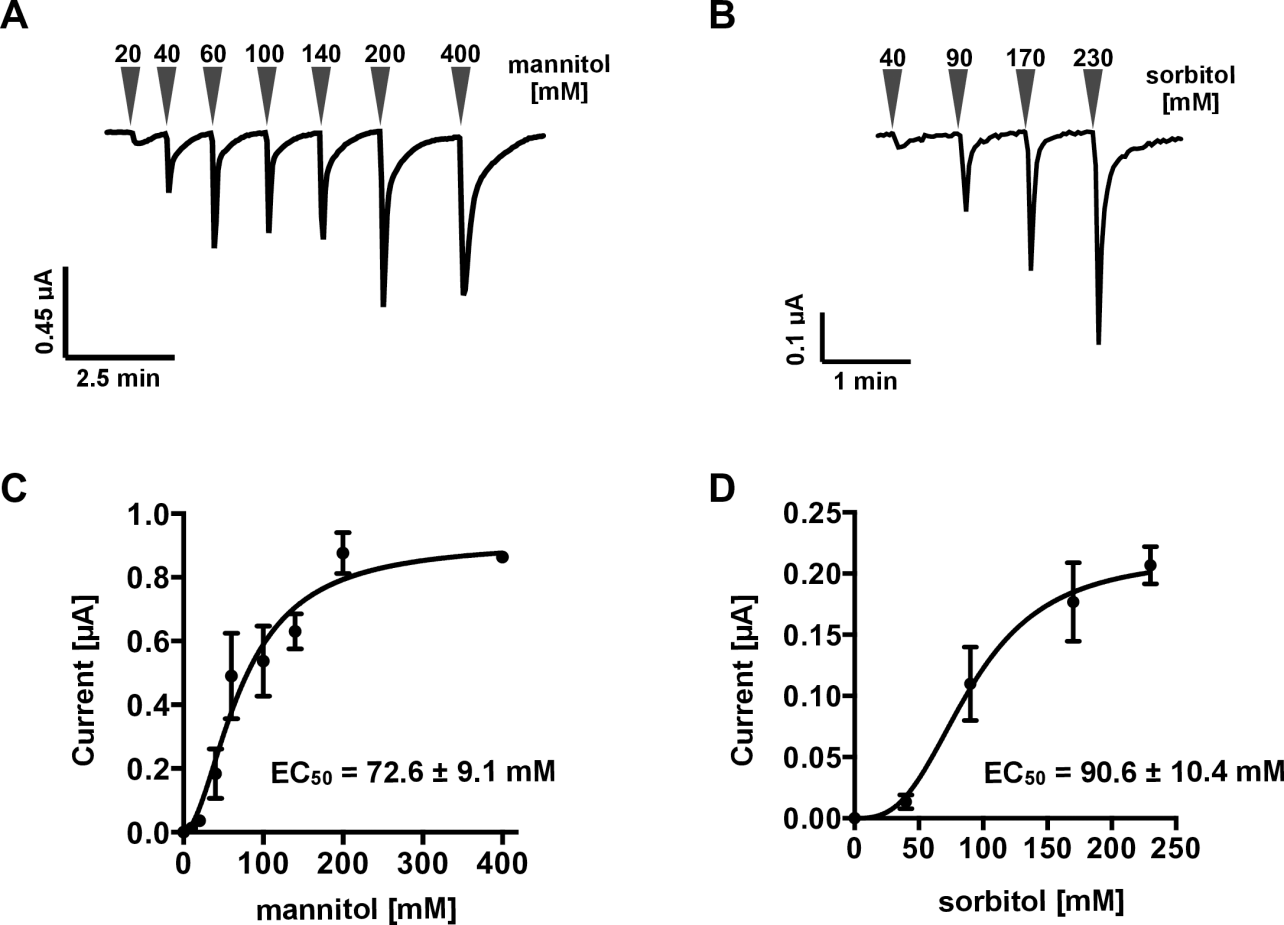
Ligand dose-dependent response of TcGr20. Two-electrode voltage clamp recordings of TcGr20-expressing *Xenopus* oocytes. A–B. Inward current response of TcGr20-expressing oocytes with a range of 20 to 400 mM mannitol (A), and 40 to 230 mM sorbitol (B). Each arrowhead represents various concentrations. C–D. Curves were fitted with a standard slope, and EC_50_ values were calculated for mannitol (C) and sorbitol (D), respectively. Data are shown as mean ± S.E.M. (n = 3).

We attempted to amplify the ORF in *TcGr20*–*28* genes for subcloning into the *Xenopus* oocyte expression vector. However, we failed to obtain PCR products from *TcGr23*, *TcGr24*, *TcGr25*, and *TcGr26* from any cDNA produced from any tissue sample. Hence, we investigated the biochemical functions of TcGr20, TcGr21, TcGr27, and TcGr28 using an electrophysiological analysis. Two-electrode voltage-clamp recording was performed as described previously [9]. We confirmed the BmGr10 response to *myo*-inositol and used this as a positive control (S1 Fig). Oocytes expressing *TcGr20*, *21*, *27*, or *28* were clamped with electrode capillaries filled with 3 M KCl. When mannitol was added to the perfusion chamber, an inward current was observed in *TcGr20*-expressing oocytes (Fig 3A), but not in water-injected or in *TcGr21*-, *TcGr27*- or *TcGr28*-expressing oocytes. (S2 Fig, Fig 3B). TcGr20 also responded to sorbitol but not to other sugars (Fig 3A). We did not observe a current response in *TcGr21*, *TcGr27* or *TcGr28*-expressing oocytes for any sugars/sugar alcohols tested. To confirm these results, we expressed *Xenopus* oocytes with *TcGr21*, *27*, or *28* fused to *AcGFP1*, or with *AcGFP1*. TcGr21-, TcGr27-, or TcGr28-AcGFP1 fluorescence was detected in the cellular membrane of oocytes, but a low level of expression was seen in oocytes injected with AcGFP1 cRNA only (S3 Fig). The mannitol/sorbitol-induced currents observed in TcGr20 expressing oocyte showed concentration-dependent responses to 20–200 mM mannitol or 40–230 mM sorbitol (Fig 4A and 4B). Based on the dose-response curves, the EC_50_ value of mannitol and sorbitol were 72.6 ± 9.1 mM and 90.6 ± 10.4 mM, respectively (Fig 4C and 4D). This result suggests that TcGr20 functions as a mannitol/sorbitol receptor.

### Tissue expression of *TcGr20*

**Fig 5:**
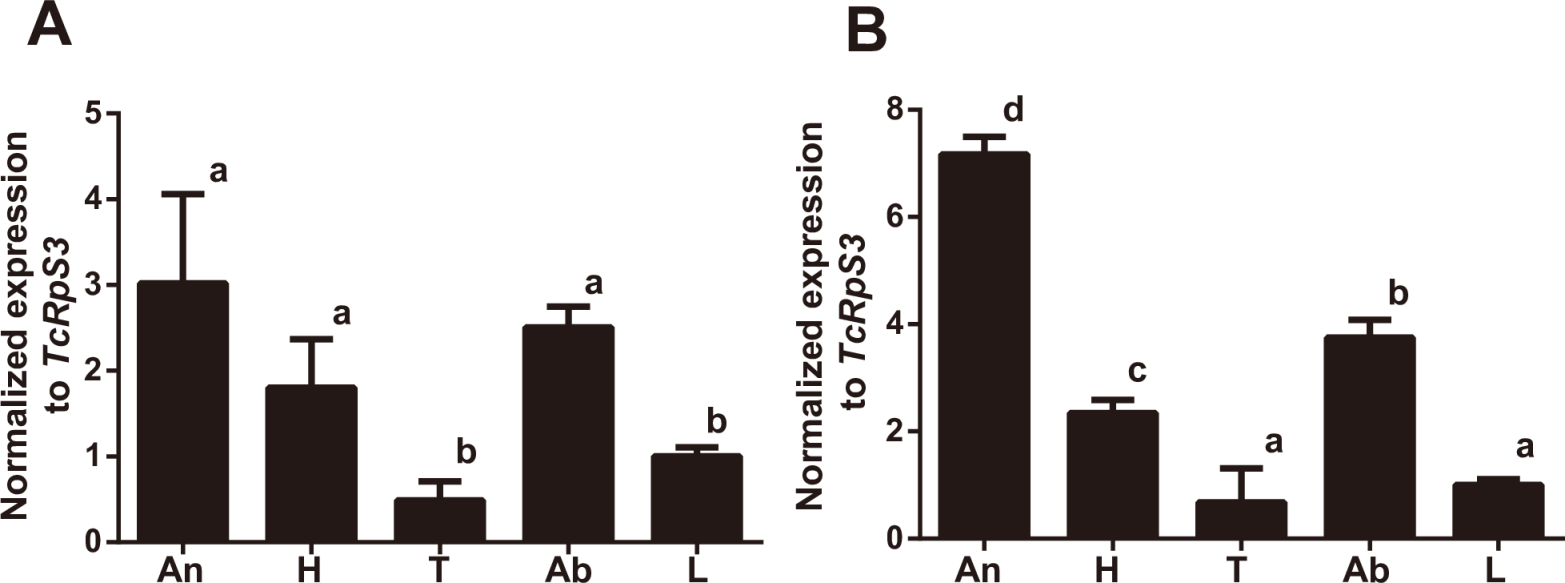
Tissue expression of *TcGr20*. The relative expression level of the *TcGr20* in different tissues was determined by quantitative RT-PCR. *TcGr20* expression in male (A) and female (B). An, antennae; H, head; T, thorax; Ab, abdomen and L, legs. Relative expression was calculated using the 2-*ΔΔ*Ct method. Ribosomal protein S3 (*RpS3*) in *T*. *castaneum* was used as the control to normalize the amount of templates. Data are shown as mean ± S.E.M. (n = 3). Statistical analyses were performed one-way ANOVA (A. *F* (DFn, DFd) *F* (4, 10) = 14.17, *P* = 0.0004; B. *F* (DFn, DFd) F (4, 10) = 163.8, *P* < 0.0001) and the post hoc Tukey’s multiple comparison test. Columns labeled with the same letters indicate not significant difference (*P* > 0.05).

Previous studies using RNA sequencing and *in situ* RT-PCR have shown that *TcGr20* is predominantly expressed in the antennae, mouthparts, head, and legs [15, 26]. Our findings here confirmed that *TcGr20* is mainly expressed in the antennae, head including the mouthparts, and abdomen in both males and females. *TcGr20* was highly expressed in antennae in both male and female adults (Fig 5A and 5B). TcGr20 on the external organs likely acts as a sensor for mannitol and sorbitol.

### Effect of mannitol/sorbitol concentrations in the dietary intake assay

**Fig 6:**
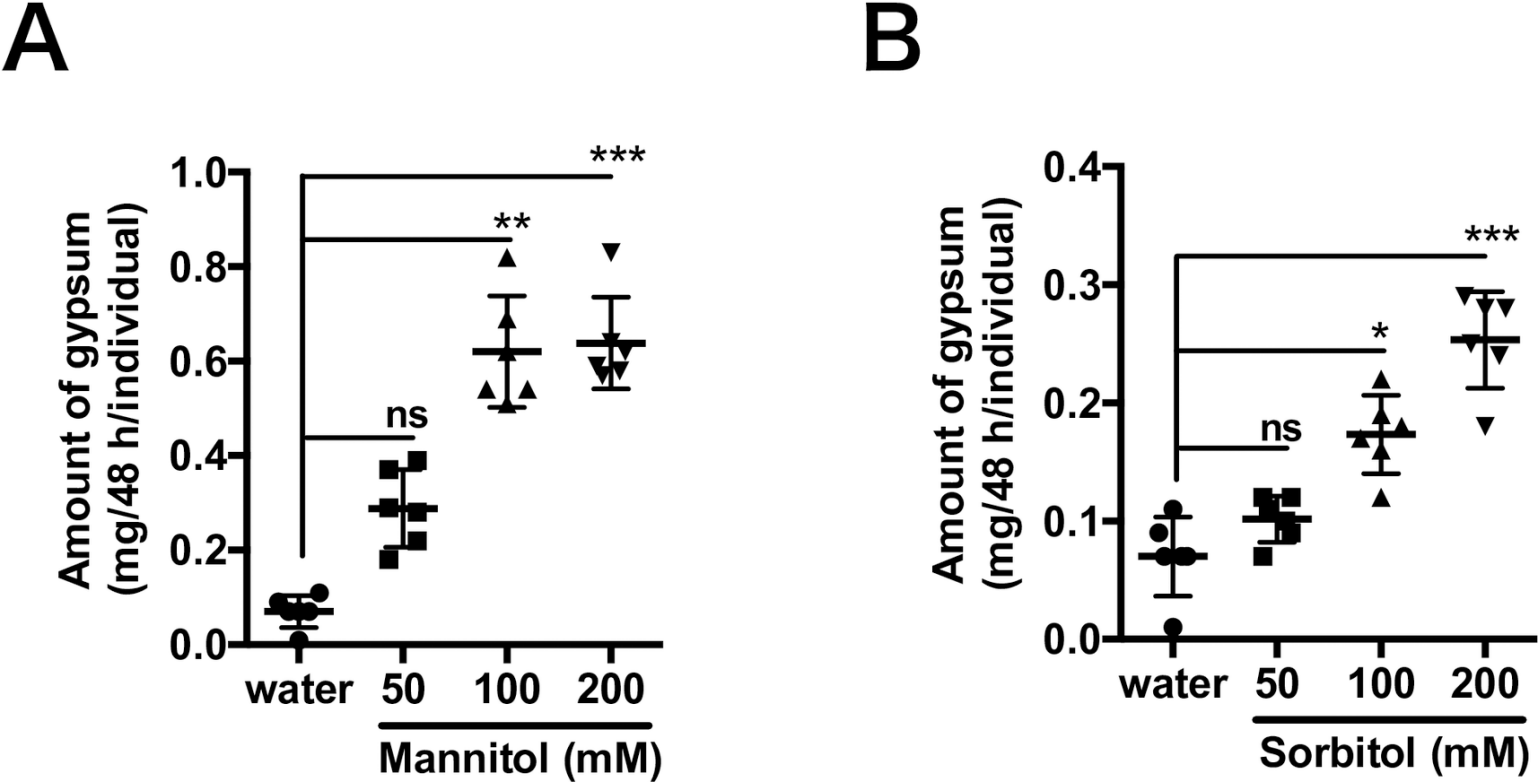
Concentration response of mannitol and sorbitol in TribUTE assay. The dose effect of gypsum intake with sugar alcohol mixture. A. mannitol; B. sorbitol. Sugar alcohols at various concentrations were contained in the gypsum block. *T*. *castaneum* adults fed on the gypsum for 48 h. Scatter plot represents the amount of excretion of adult beetles in individuals (n = 6). Standard error bars show S.E.M. The results presented are representative of three separate experiments. Statistical analyses were performed Kruskal-Wallis test (A. “***” *P* = 0.0002, n = 24; B. “***” *P* = 0.0002, n = 24) and Dunn's multiple comparisons test (“*” *P* ≤ 0.05, “**” *P* ≤ 0.01, “***” *P* ≤ 0.001, “ns” not significant).

We measured the dietary intake of *T*. *castaneum* adult beetles using gypsum containing mannitol or sorbitol at various concentrations (Fig 6). Dietary intake increased in the presence of 100 mM mannitol (Fig 6A) and 100 mM sorbitol (Fig 6B). The amount of gypsum excreta in individuals at 48 h was 0.62 ± 0.05 mg for 100 mM mannitol, and 0.17 ± 0.01 mg for 100 mM sorbitol (Fig 6). These results indicate that mannitol stimulates a feeding response even at low concentrations and promotes dietary intake by the beetles.

### Evaluation of dietary intake in *TcGr20*-silencing *T*. *castaneum*

**Fig 7:**
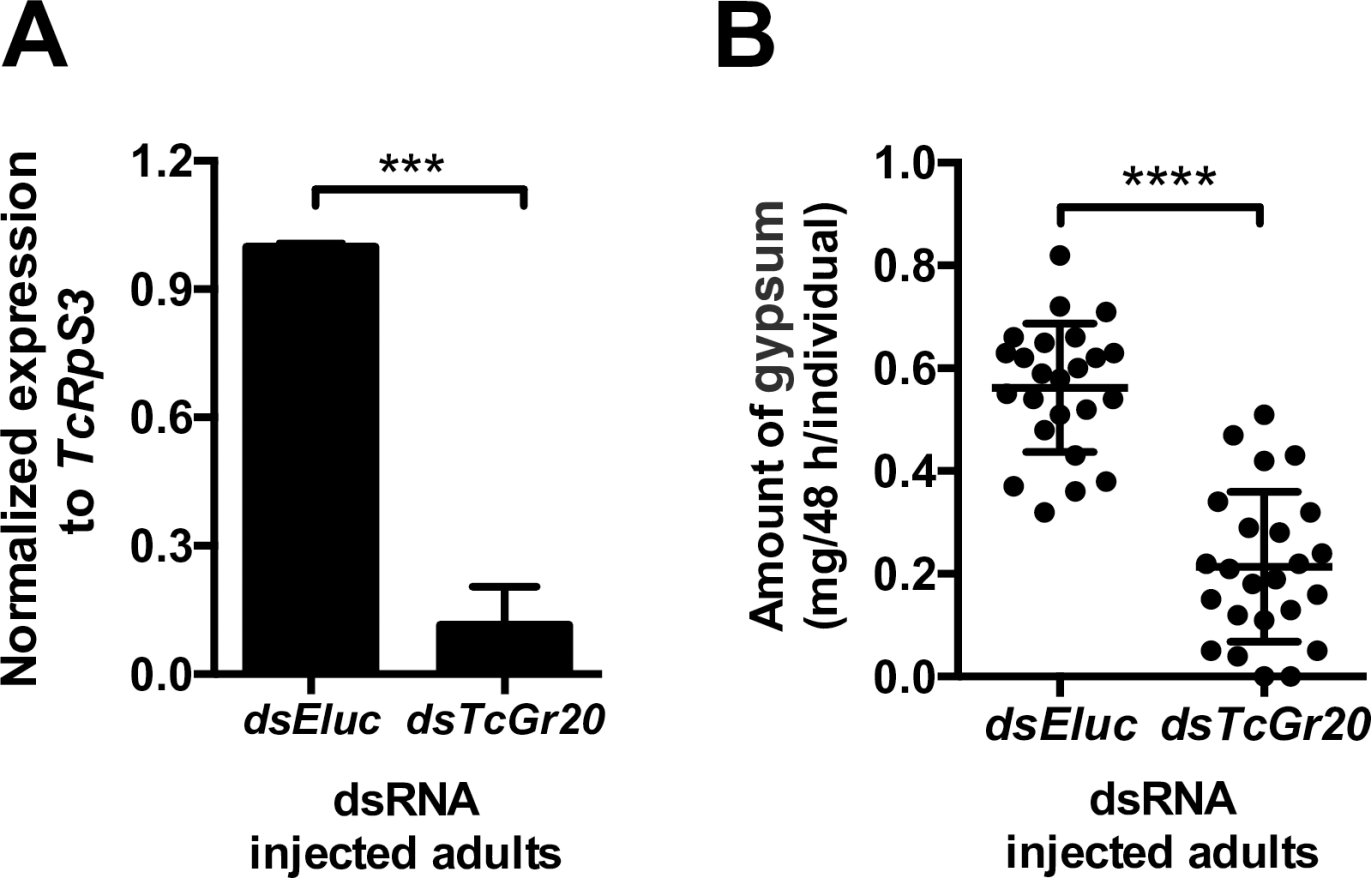
TribUTE assay in *TcGr20*-silencing *T*. *castaneum*. A. Knockdown of *TcGr20* using the injection of the dsRNA into the starved adult beetles. The *Eluc*-dsRNA was injected as a control. *TcGr20* expression levels of whole body were examined at 48 h after the dsRNA injection. Standard error bars show S.E.M. Statistical significance was determined by *t*-test (*P* = 0.0006). B. Effect of gypsum intake supplemented with 100 mM mannitol. The gypsum in the presence of 100 mM mannitol was given to the *TcGr20*- and *Eluc*-dsRNA-injected adult beetle individuals at 48 h after dsRNA injection, respectively for 48 h. The amount of excreta was measured using microbalance. Each plot represents the amount of excreta of adult beetles in individuals (n = 24). Standard error bars show S.E.M. Statistical significance was determined by *t*-test (“****” *P* ≤ 0.0001).

The results described above showed that *T*. *castaneum* increased the dietary intake of gypsum supplemented with mannitol (Fig 6A), and that TcGr20 was a mannitol receptor (Fig 3A). We therefore investigated whether TcGr20 was involved in mannitol recognition for feeding. We used RNA interference (RNAi) approach to validate gene function, as this was shown previously to be effective in *T*. *castaneum* [27]. We injected *TcGr20* double-strand RNA (dsRNA) into *T*. *castaneum* adults. As a result, *TcGr20* expression was significantly reduced compared with that of *emerald luciferase (Eluc)-*dsRNA injected adults (Fig 7A). Using the *TcGr20* dsRNA-injected adult beetles, we evaluated the dietary intake of gypsum in the presence of with 100 mM mannitol. The amounts of excreta from *TcGr20* dsRNA-injected adults significantly decreased in the presence of 100 mM mannitol compared to that of *Eluc* dsRNA-injected adults (Fig 7B). This result indicates that *TcGr20* RNAi was an effective approach, and that TcGr20 was responsible for mannitol recognition *in vivo*.

## Discussion

Our results demonstrate that mannitol prolongs feeding behavior in *T*. *castaneum* adult beetles, and that TcGr20 is responsible for mannitol/sorbitol recognition and the promotion of dietary intake.

Gustatory receptor (Gr)-expressing chemosensory organs are involved in external non-volatile compound recognition. The antennae, mouthparts, and legs of *Tribolium* recognize sucrose in electrophysiological analyses [15, 28]. In *B*. *mori*, the *myo*/*epi*-inositol receptor BmGr10, which belongs to the Gr43-like family, on chemosensory organs recognizes sugar alcohols [9]. Hence, TcGrs located on the antennae, mouthparts, and legs were hypothesized to be involved in the perception of mannitol as an external signal. Previous analyses such as tissue RNA-seq and *in situ* PCR have also shown that *TcGr20* is expressed in the antennae, mouthparts, head, legs, and body [15, 26], and our results are consistent with these findings. The taste sensilla on the legs may be able to assess gypsum blocks supplemented with sweeteners [29, 30]. *TcGr20* expression was observed in the abdomen. Similar results were also obtained in *Drosophila*, where *DmGr* genes were shown to be expressed in midgut cells and multidendritic sensory neurons [30, 31]. TcGr20 may have similar functions in these tissues. In a previous work, differences in *Gr* expression levels between males and females were observed [32], implying the occurrence of sex-specific feeding behavior. However, we observed no difference in *TcGr20* expression and also no sex-related differences in the dietary intake assays (Fig 5, S5 Fig).

Electrophysiological analyses using *Xenopus* oocytes showed that TcGr20 contributes to responses to mannitol and sorbitol (Fig 3). The EC_50_ values of TcGr20 for these sugar alcohols showed that the mannitol response was induced by a 0.8-fold lower concentration than the sorbitol response. Additionally, the dietary intake of *T*. *castaneum* adult beetles in the TribUTE assay was more sensitive to mannitol than sorbitol (Fig 6). The amount of excreta when *T*. *castaneum* beetles were given gypsum in the presence of 100 mM mannitol was markedly greater than after supplementation with 100 mM sorbitol (Fig 6), indicating that the concentration response of mannitol appears to be correlated with that of TcGr20 response levels in the electrophysiological analysis. It is likely that TcGr20 mainly regulates mannitol recognition in the gustatory organs. We also examined whether TcGr20 is involved in mannitol recognition using RNAi and the TribUTE assay. We found that *TcGr20* expression levels after eclosion in *TcGr20*-dsRNA-injected pupae showed a negligible difference compared to those injected with *Eluc*-dsRNA (S4 Fig). The 6–9-day period of pupal development prior to adulthood [33] may have decreased the gene silencing effect. To test this possibility, we attempted dsRNA injection into starved adult beetles. The test showed a significant RNAi silencing effect (Fig 7A). Using the *TcGr20* RNAi beetles (Fig 7A), we showed that TcGr20 was responsible for mannitol recognition-dependent dietary intake behavior (Fig 7B). The activation of intake of gypsum by the *TcGr20*-dsRNA-injected beetles appeared to decrease in the presence of 100 mM mannitol, resulting in a decrease in gypsum intake. It is important to note that the silencing effects of RNAi were obtained temporarily. TcGr20 responds to both mannitol and sorbitol, but this dietary intake assay needs to be further confirmed using other methods such as the clustered regularly interspaced short palindromic repeats (CRISPR)/Cas9 system.

In *T*. *castaneum*, 8 *TcGr* genes in the *Tribolium* genome (NCBI public data, ID: 216) were found as the Gr43-like clade (Fig 2). This study demonstrated that TcGr20 was a mannitol/sorbitol receptor, but the functions of other *TcGr* genes remain unclear, as we could not amplify PCR products for *TcGr23*, *TcGr24*, *TcGr25*, and *TcGr26* from any cDNA. The 7 *TcGr* genes would allow *T*. *castaneum*, as a generalist feeder, to be capable of recognizing a large number of natural attractants such fructose and sugar alcohols. Since these *TcGr* genes are highly expressed in the antennae and mouthparts, and contribute to the beetles’ ground-dwelling life style and scanning behavior [15], they may respond to non-volatile stimuli produced by host plants. As alternative candidate targets, we expected that compounds produced by fungi would be attractive, since beetles are strongly attracted to chemical stimuli from fungi grown on flour and cotton seeds [34–37]. When the beetle larvae fed on *Aspergillus niger*, suitable development occurs and the reproductive potential of females eventually increases [34–36].

Mannitol is the most widely distributed of polyols and is found in more than 50 species of plants, algae, fungi, and lichens [38, 39]. In particular, dried seaweeds contain 1–1.7 M mannitol as a major carbohydrate component [40]. Sorbitol is also present in prunes at 2.4 g/100 g dry weight and pears at 4.6 g/100 g dry weight [41]. *T*. *castaneum* can recognize these stored foods as the sugar alcohols are present at much higher concentrations than are required for response by TcGr20. *A*. *niger* produces 45–210 mM mannitol [42], suggesting that *T*. *castaneum* might be able to recognize the fungus via TcGr20. Fungal growth on stored products and bulk grains is favored by high-moisture conditions. It is possible *T*. *castaneum* may be attracted to such stored products using moisture and fungal attractants as cues. *T*. *castaneum* is also a harmful wheat flour pest [12, 43] and attacks cake flour and whole-wheat flour, which contain approximately 0.032 mg/g and 0.01 mg/g dry weight mannitol, respectively (S5 Table); these levels are far lower than biochemically required for TcGr20 recognition. Therefore, *T*. *castaneum* adults are unlikely to recognize wheat flour using TcGr20. Rather, they more likely recognize sugars such as glucose and fructose contained in the wheat flour. It is possible that *T*. *castaneum* may also recognize volatiles emitted by wheat flour using olfactory receptors, but this has yet to be confirmed.

The novel artificial dietary method described here for *T*. *castaneum* adult beetles facilitates examination of their dietary intake based on measurement of excreta. Gypsum (calcium sulfate dihydrate) is an inorganic compound that can be formed into a solid pellet in combination with water (PubChem database, CID: 24928). The observation of gypsum intake by *T*. *castaneum* demonstrated a series of behaviors from recognition and swallowing to excretion. The TribUTE assay can quantify beetle preferences for non-volatile compounds by measuring gypsum excreta without consideration of odorant stimulation. Sugars/sugar alcohols act as feeding behavior-facilitating factors in some insects [44–46], and *T*. *castaneum* significantly preferred gypsum containing these additives (Fig 1E). Our TribUTE assay demonstrated that adult beetles could, to some degree, recognize sugars such as fructose, maltose, and sucrose (Fig 1E). Based on these findings, we suggest that TcGr4–19 in the sugar clade [15] are potential candidates for recognition of these carbohydrates. Although these sugar receptors have been identified in the *T*. *castaneum* genome, their functions remain unclear. The TribUTE assay enabled the identification of non-volatile compounds associated with the food preference of *T*. *castaneum*. Notably, adult beetles can feed on gypsum without sweeteners (Fig 1). Thus, the TribUTE assay would be also applicable for exploring non-volatile compounds that induce a decrease in dietary intake by *T*. *castaneum*.

## Supporting information

S1 Table Primers for *Xenopus* oocyte expression. (PDF)

S2 Table Primers for AcGFP1 fusion expression. (PDF)

S3 Table Primers for quantitative RT-PCR. (PDF)

S4 Table Primers for double strand RNA synthesis. (PDF)

S5 Table Mannitol in flours. (PDF)

S1 Fig The BmGr10 response to *myo*-inositol as a positive control in a two-electrode voltage clamp. The current was recorded in BmGr10-expressing oocytes using the twoelectrode voltage clamp. Arrowheads represent at the point of myo-inositol addition, the concentrations in the perfusion chamber were at 50 mM.
(PDF)

S2 Fig Current recordings of *Xenopus* oocytes expressing TcGr21, TcGr27 and TcGr28 against sugars and sugar alcohols. Inward current response of *Xenopus* oocytes expressing each TcGr to candidate tastants (arrowheads). Tastants were tested at 200 mM. The current data are representative of recordings independently performed in several times. A, TcGr21; B, TcGr27; C,TcGr28.
(PDF)

S3 Fig TcGr21-, TcGr27- and TcGr28-AcGFP1 expression in *Xenopus* oocyte. The cRNA encoding TcGr21, TcGr27 and TcGr28 fused to AcGFP1 was injected into *Xenopus* oocytes. The cryo-sectionings were obtained at 3 days after injections. The AcGFP1 fluorescences were detected in the cellular membranes. Scale bars show 12 μm.
(PDF)

S4 Fig Gene silencing effect. *TcGr20* dsRNA was injected into the newly emerged pupae. *Eluc*-dsRNA was injected as a control. The injected pupae were kept at 25°C until the emergence of adult beetles. The *TcGr20* expression levels of whole body were examined after eclosion. Statistical significance was determined by t-test. Relative expression is shown in 2-*ΔΔ*Ct method. Ribosomal protein S3 (*RpS3*) in *T*. *castaneum* was used as the normalized control. Data are shown as mean ± S.E.M. (n = 3).
(PDF)

S5 Fig The effect of gypsum intake between male and female. *T*. *castaneum* adult beetles were individually fed on gypsum blocks in the presence of 200 mM mannitol for 48 h. The amount of excreta was measured using microbalance. Each plot represents the amount of excreta of adult beetles in individuals (n = 18). Standard error bars show S.E.M. Statistical analyses were performed Mann-Whitney U test (P = 0.87). “ns” no significant. Total numbers are 36.
(PDF)

S1 File Supplemental procedure. (PDF)

S2 File Raw data. (XLSX)

## Abbreviations

ORF: open reading frame
RT: reverse transcription
RNAi: RNA interference
dsRNA: double strand RNA
cRNA: capped RNA
MBS: modified Barth’s saline
CBB: Coomassie brilliant blue
Gr: gustatory receptor
