# Supplementary material for: A mannitol/sorbitol receptor stimulates dietary intake in Tribolium castaneum
Source: PLoS One. 2017 Oct 12;12(10):e0186420. doi: 10.1371/journal.pone.0186420 (PMC5638539; doi:10.1371/journal.pone.0186420)
Supplement: S1 Table — (PDF) [file pone.0186420.s001.pdf]

S1 Table Primers for *Xenopus* oocyte expression

Gene	NCBI ID	Amplicon (bp)	Name	Direction	Sequence	Restriction enzyme
TcGr20	XM_015980833.1	1218	TcGr20_f1	forward	CTCagatctCCACCATGAGTGCAATGTCGACGGT	BglII
			TcGr20_r1	reverse	CTTgatatcTTAATTAAACTGAAACAAAATCACT	EcoRV
TcGr21	XM_008195143.2	1155	TcGr21_f1	forward	CTCagatctCCACCATGAGTGCAATGTCGACGGT	BglII
			TcGr21_r1	reverse	CTTgatatcTTAATTAAACTGAAACAAAATCACT	EcoRV
TcGr23	XM_015983119.1	1134	TcGr23_f1	forward	CTCagatctATGACTATCACCATATCTAAAAC	BglII
			TcGr23_r1	reverse	CTTgatatcTCAACCGTTTTTATTAAACTGAA	EcoRV
TcGr24	XM_015980720.1	1239	TcGr24_f1	forward	CTCagatctATGCATCGAACGGAAATTCCT	BglII
			TcGr24_r1	reverse	CTTgatatcTTAGTCGCGGGAGTAGTAGA	EcoRV
TcGr25	XM_015980725.1	1191	TcGr25_f1	forward	CTCagatctCCACCATGTTTCGTGACATCACACTC	BglII
			TcGr25_r1	reverse	CTTgatatcTTAAAGTTTTGTAAACTGTATAAAAA	EcoRV
TcGr26	XM_015984769.1	1221	TcGr26_f1	forward	CTCagatctCCACCATGTATACTGTAATTTCCCCCC	BglII
			TcGr26_r1	reverse	CTTgatatcTCATATTTTGCTTGTTCTTGAGC	EcoRV
TcGr27	XM_015984763.1	1221	TcGr27_f1	forward	CTCagatctCCACCATGACAAAGTTTCCCGCAATTA	BglII
			TcGr27_f1	reverse	CTTgatatcTTACACCACTTGTATTGTTCTG	EcoRV
TcGr28	XM_015980984.1	1179	TcGr28_f1	forward	CTCagatctCCACCATGACCAGTAGTAAACCATTGT	BglII
			TcGr28_r1	reverse	CTTgatatcTTACACCACTTGTATTGTTCTG	EcoRV
