# Supplementary material for: A mannitol/sorbitol receptor stimulates dietary intake in Tribolium castaneum
Source: PLoS One. 2017 Oct 12;12(10):e0186420. doi: 10.1371/journal.pone.0186420 (PMC5638539; doi:10.1371/journal.pone.0186420)
Supplement: S2 Table — (PDF) [file pone.0186420.s002.pdf]

S2 Table Primer for AcGFP1 fusion expression

	Forward	Reverse
TcGr21	AGTTTAAAATGGTGAGCAAGGGCGCC	CTCACCATTTTAAACTGAAACAAAATGACTAA
TcGr27	AAGTGGTGATGGTGAGCAAGGGCGCC	CTCACCATCACCACTTGTATTGTTCTGGA
TcGr28	CTGGATTGATGGTGAGCAAGGGCGCC	CTCACCATCAATCCAGTTGTACTAGCCC
pT7XbG2 vector	ACAAGTGAGATATCGAATTCGGATCTGG	TCGATATCTCACTTGTACAGCTCATCCAT
