# Supplementary material for: A mannitol/sorbitol receptor stimulates dietary intake in Tribolium castaneum
Source: PLoS One. 2017 Oct 12;12(10):e0186420. doi: 10.1371/journal.pone.0186420 (PMC5638539; doi:10.1371/journal.pone.0186420)
Supplement: S3 Table — (PDF) [file pone.0186420.s003.pdf]

S3 Table Primers for quantitative RT-PCR

Name	Direction	Sequence
RpS3_f1	forward	TGGCGATGGCGTTTTCAAAG
RpS3_r1	reverse	ATGATTTCCGTGCGAGTTGG
TcGr20_f2	forward	AATGATTCCTTTGGGTTGCTG
TcGr20_r2	reverse	GGTAAATATAATGGCGTAAAGG
