# Supplementary material for: A mannitol/sorbitol receptor stimulates dietary intake in Tribolium castaneum
Source: PLoS One. 2017 Oct 12;12(10):e0186420. doi: 10.1371/journal.pone.0186420 (PMC5638539; doi:10.1371/journal.pone.0186420)
Supplement: S4 Table — (PDF) [file pone.0186420.s004.pdf]

S4 Table Primers for double strand RNA synthesis

Name	Direction	Sequence
TcGr20_f3	forward	TAGGGCGATGTCGACGGTTATAGTGTCA
TcGr20_r3	reverse	TACCCAATGCTCTGATTTCGCGCAATTAA
rTcGr20_f3	forward	TAGGGCGAACAGCTGCCAATATCACAG
rTcGr20_r3	reverse	TACCCAATCGAGACTAAAGCGCGTTAA
Eluc_f3	forward	TAATACGACTCACTATAGGGTCCTGGTCTTCACCACTAG
Eluc_r3	reverse	TCCTGGTTAAACCTTCGGAG
rEluc_f3	forward	TAATACGACTCACTATAGGGTCCTGGTTAAACCTTCGGAG
rEluc_f3	reverse	TCCTGGTCTTCACCACTAGG
