# Supplementary material for: A mannitol/sorbitol receptor stimulates dietary intake in Tribolium castaneum
Source: PLoS One. 2017 Oct 12;12(10):e0186420. doi: 10.1371/journal.pone.0186420 (PMC5638539; doi:10.1371/journal.pone.0186420)
Supplement: S5 Table — (PDF) [file pone.0186420.s005.pdf]

S5 Table Mannitol in flours (mg/g [w/w])

	sample 1	sample 2	sample 3	Average ± S.D.*	Supplier
cake flour	0.029	0.032	0.034	0.032 ± 0.002	AEON Co., Ltd., Chiba, Japan
whole-wheat flour	0.008	0.011	0.01	0.01 ± 0.002	Pioneer-kikaku, Kanagawa, Japan

*S.D. indicates "standard deviation".
