# Supplementary figures and images for: A mannitol/sorbitol receptor stimulates dietary intake in Tribolium castaneum
Source: PLoS One. 2017 Oct 12;12(10):e0186420. doi: 10.1371/journal.pone.0186420 (PMC5638539; doi:10.1371/journal.pone.0186420)

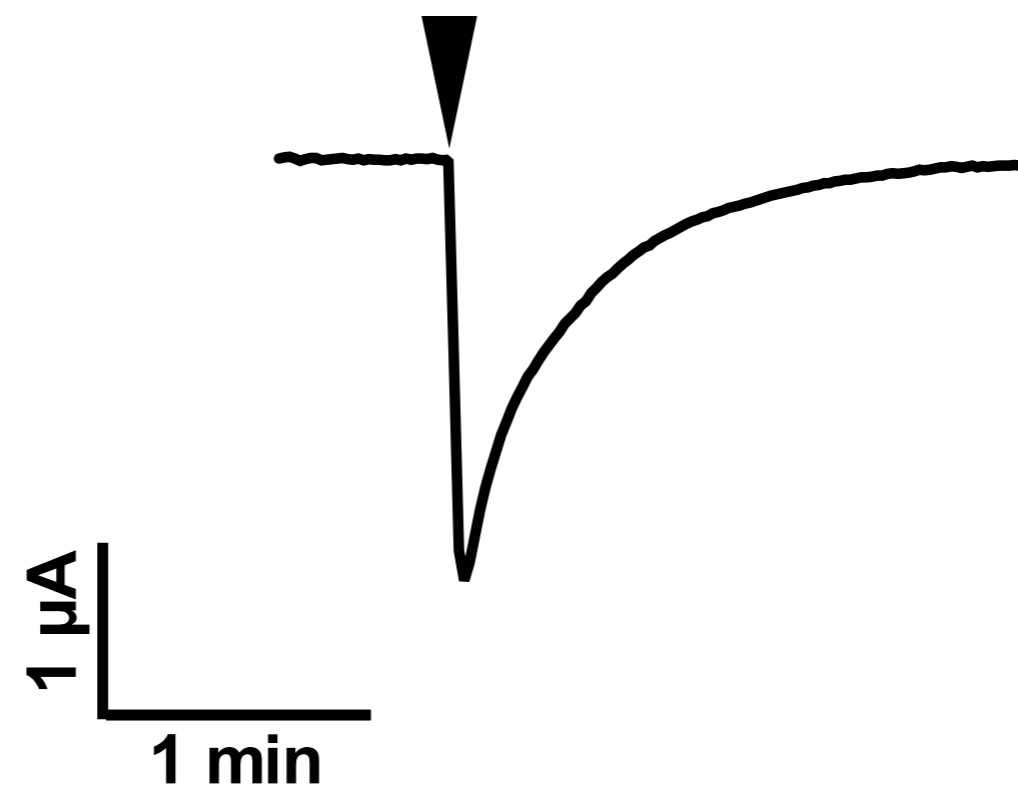

Supplement: S1 Fig — The current was recorded in BmGr10-expressing oocytes using the twoelectrode voltage clamp. Arrowheads represent at the point of myo-inositol addition, the concentrations in the perfusion chamber were at 50 mM. (PDF) [file pone.0186420.s006.pdf]

A

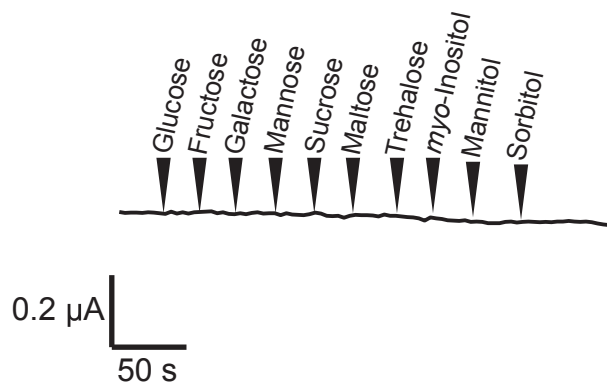

B

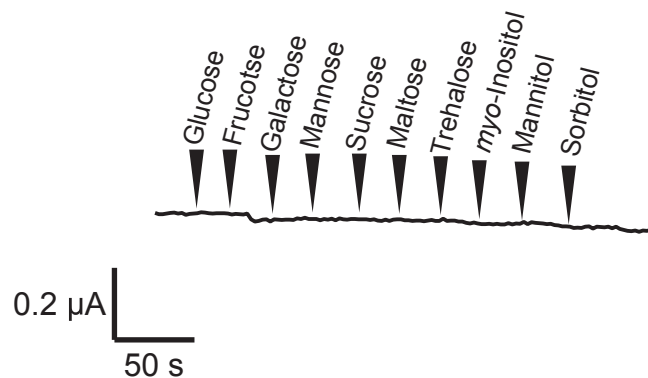

C

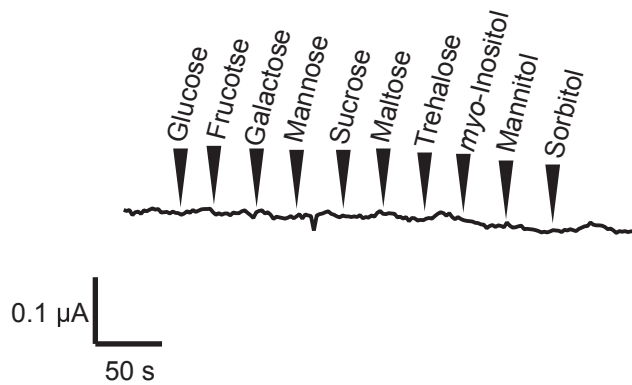

Supplement: S2 Fig — Inward current response of Xenopus oocytes expressing each TcGr to candidate tastants (arrowheads). Tastants were tested at 200 mM. The current data are representative of recordings independently performed in several times. A, TcGr21; B, TcGr27; C,TcGr28. (PDF) [file pone.0186420.s007.pdf]

TcGr21-AcGFP1

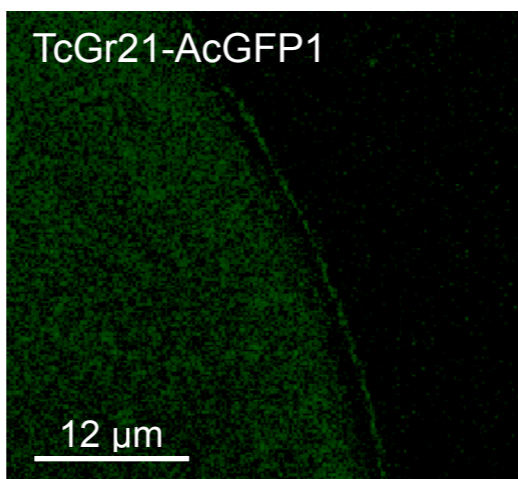

TcGr27-AcGFP1

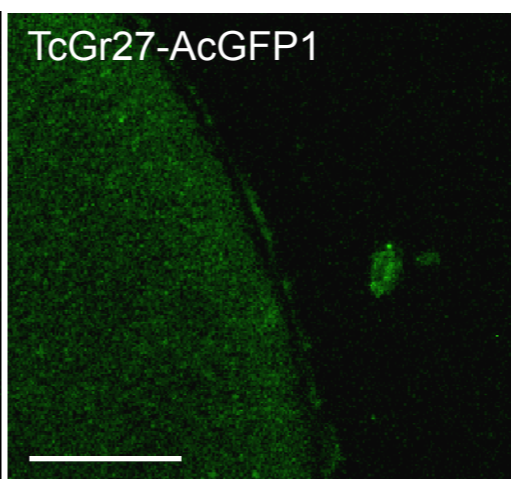

TcGr28-AcGFP1

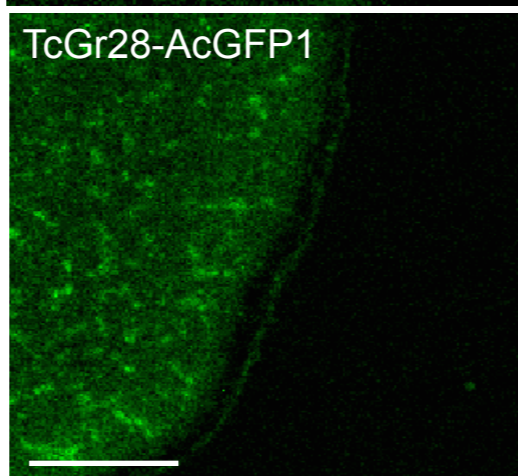

AcGFP1

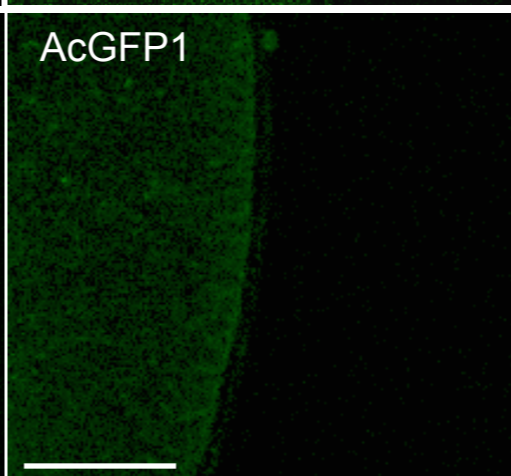

Supplement: S3 Fig — The cRNA encoding TcGr21, TcGr27 and TcGr28 fused to AcGFP1 was injected into Xenopus oocytes. The cryo-sectionings were obtained at 3 days after injections. The AcGFP1 fluorescences were detected in the cellular membranes. Scale bars show 12 μm. (PDF) [file pone.0186420.s008.pdf]

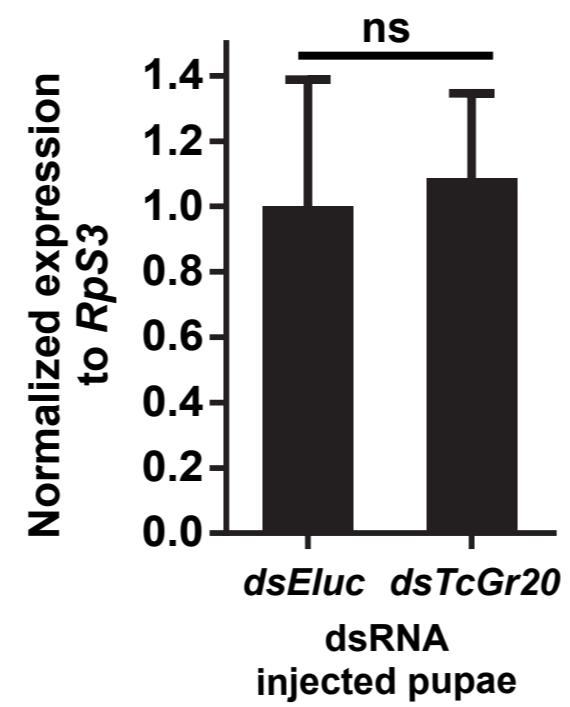

Supplement: S4 Fig — TcGr20 dsRNA was injected into the newly emerged pupae. Eluc-dsRNA was injected as a control. The injected pupae were kept at 25°C until the emergence of adult beetles. The TcGr20 expression levels of whole body were examined after eclosion. Statistical significance was determined by t-test. Relative expression is shown in 2-ΔΔCt method. Ribosomal protein S3 (RpS3) in T. castaneum was used as the normalized control. Data are shown as mean ± S.E.M. (n = 3). (PDF) [file pone.0186420.s009.pdf]

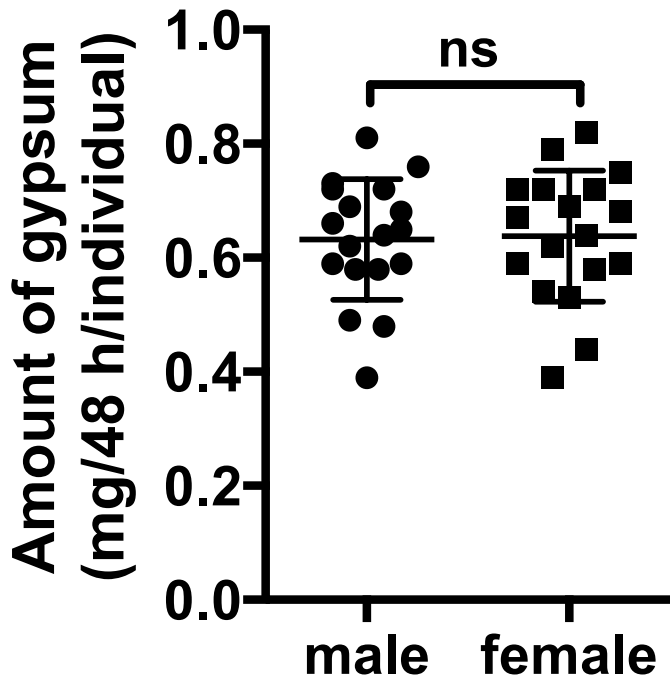

Supplement: S5 Fig — T. castaneum adult beetles were individually fed on gypsum blocks in the presence of 200 mM mannitol for 48 h. The amount of excreta was measured using microbalance. Each plot represents the amount of excreta of adult beetles in individuals (n = 18). Standard error bars show S.E.M. Statistical analyses were performed Mann-Whitney U test (P = 0.87). “ns” no significant. Total numbers are 36. (PDF) [file pone.0186420.s010.pdf]
