# Supplementary material for: A mannitol/sorbitol receptor stimulates dietary intake in Tribolium castaneum
Source: PLoS One. 2017 Oct 12;12(10):e0186420. doi: 10.1371/journal.pone.0186420 (PMC5638539; doi:10.1371/journal.pone.0186420)
Supplement: S1 File — (PDF) [file pone.0186420.s011.pdf]

Supplementary procedure

Transient expression of AcGFP1 fused TcGrs in *Xenopus* oocytes

AcGFP1 was introduced into pT7XbG2-TcGr21, 27 and 28 *Xenopus* oocyte expression vector using GeneArt® Seamless Cloning and Assembly kit (Thermo Fisher Scientific). The inverse PCR reactions were performed using PrimeSTAR® Max DNA Polymerase (TaKaRa Bio). The open reading frame of AcGFP1 was amplified from pT7XbG2-AcGFP1 (DDBJ accession number, AB255038) vector using high-fidelity PCR polymerase, PrimeSTAR® HS (TaKaRa Bio). The primer sequences were shown in S2 Table. The stop codons of TcGr21, 27 and 28 were excluded by PCR reactions. Sequence analyses were performed by eurofins Genomics (Tokyo, Japan) to confirm the correctness of the construct. Sequence data were analyzed using FinchTV sequence scanner software. Capped RNA (cRNA) synthesis was performed in accordance with the manufacturer's instructions. The expression procedures were referred to materials and methods. The cRNA injected *Xenopus* oocytes were kept at 20°C in darkness for 3 days. The procedure from the obtainment of frozen sections to the observation was described in [ref 9].

Quantification of mannitol content in wheat flours

Commercially available wheat flours were put into deionized water in a ratio of 2:5 (w/w), and were thoroughly vortexed for 2 min. The suspensions were centrifuged at 10000 G for 10 min at 25°C. The supernatant was used for the quantification using EnzyChrom™ D-Mannitol Assay Kit (BioAssay Systems, San Francisco, CA) based on colorimetry. The measurements were examined according to the manufacturer’s instructions. Optical density at 570 nm was measured using microplate-reader, iMark™ (Bio-Rad, Hercules, CA) at 25°C.
